# Depleting the 19S proteasome regulatory PSMD1 subunit as a cancer therapy strategy

**DOI:** 10.1002/cam4.5775

**Published:** 2023-03-19

**Authors:** Julia Adler, Roni Oren, Yosef Shaul

**Affiliations:** ^1^ Department of Molecular Genetics Weizmann Institute of Science Rehovot Israel; ^2^ Department of Veterinary Resources Weizmann Institute of Science Rehovot Israel

**Keywords:** 19S regulatory particle, 26S proteasome, breast cancer, cancer therapy, mouse xenografts, ovarian cancer PDX

## Abstract

**Background:**

Proteasome inhibitors are in use in treating certain types of cancers. These drugs inhibit the catalytic activity of the 20S proteasome, shared by all the different proteasome complexes. Inhibitors of the 26S‐associated deubiquitinating activity explicitly inhibit the 26S proteasomal degradation of ubiquitinylated substrates. We have previously reported an alternative strategy that is based on reducing the 26S/20S ratio by depleting PSMD1, 6, and 11, the subunits of the 19S proteasome regulatory complex. Given the addiction of the many cancer types to a high 26S/20S ratio, the depletion strategy is highly effective in killing many aggressive cancer cell lines but not mouse and human immortalized and normal cells.

**Methods:**

We used two aggressive cell lines, MDA‐MB‐231, a triple‐negative breast tumor cell line, and OVCAR8, a high‐grade ovary adenocarcinoma. Cell culture, mouse MDA‐MB‐231, OVCAR8 xenografts, and patient‐derived ovarian cancer xenograft (PDX) models were transduced with lentivectors expressing PSMD1 shRNA. Tumor size was measured to follow treatment efficacy.

**Results:**

Using different experimental strategies of expressing shRNA, we found that PSMD1 depletion, either by expressing PSMD1 shRNA in an inducible manner or in a constitutive manner, robustly inhibited MDA‐MB‐231, and OVCAR8 xenograft tumor growth. Furthermore, the PSMD1 depletion strategy compromised the growth of the PDX of primary ovarian cancer.

**Conclusion:**

Our results suggest that reducing the 26S/20S ratio might be a valuable strategy for treating drug‐resistant aggressive types of cancers.

## Graphical abstract

Depletion of the 26S proteasome but not the 20S proteasome robustly inhibits the growth of triple‐negative breast cancer and ovarian cancer cell lines in xenograft models.

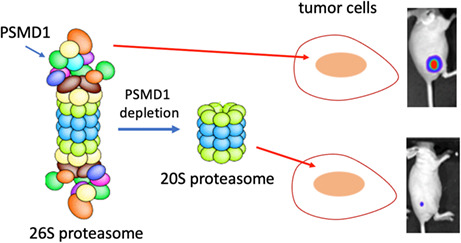

## 1 INTRODUCTION

Proteasomes play crucial roles in maintaining cellular physiology and homeostasis.^1^ Two major proteasome complexes (PCs), 26S and 20S, are active in the cells. Tumors express high levels of proteasome subunits and display higher proteasome activity.^2^, ^3^, ^4^ Ras‐transformed human and mouse immortalized cells show an increased level of the 26S proteasome subunits and 26S complex.^4^ Cancer cells exhibit high sensitivity to proteasome inhibition.^5^, ^6^ Proteasome inhibitors are utilized in treating lymphoid malignancies, particularly multiple myeloma.^7^, ^8^ Proteasome inhibitors are also efficient in killing solid and hematologic tumors.^5^, ^9^ Bortezomib, MG132, and carfilzomib inhibit the catalytic activity of the 20S PC in inhibiting all the different proteasome types, including 26S.^10^ The proteasomal pan‐inhibition might explain the peripheral neuropathy and neuromuscular and cardiovascular adverse effects.^11^, ^12^

The 26S proteasome comprises the 20S catalytic domain and one or two 19S regulatory particles (RP).^13^, ^14^, ^15^ The 19S RP is formed by two distinct complexes: the base and the lid. The base is composed of six paralogous AAA‐ATPases termed PSMC1–PSMC6 and three non‐ATPases, PSMD2, PSMD1, and ADRM1. The lid has nine subunits: PSMD3, 6, 7, 8, 11, 12, 13, 14, and SHFM1 (DSS1).

The poly‐ubiquitinated proteins are the major 26S proteasome substrates.^1^ The ubiquitylated substrates interact, via protein–protein interaction, with specific subunits of the 19S RP of the 26S proteasome. The substrate is then deubiquitinated, unfolded by the ATPases, and translocated into the 20S catalytic chamber for degradation.^13^, ^16^, ^17^ The described multistep process of protein degradation path is the avenue for alternative approaches to blocking proteasomal protein degradation. These include a specific PSMD14 inhibitor, the 19S RP‐associated deubiquitinating enzymes,^18^ and proteasome‐ubiquitin receptor ADRM1.^19^

The 26S/20S proteasome ratio is a dynamic process and tightly regulated. A typical resistance mechanism to proteasome inhibitors is mediated by the reduced cellular 26S/20S proteasome ratio.^20^, ^21^ Thus, the ratio of PCs regulates cell homeostasis. The transformation process leads to an increased dependency on proteasome function as part of the global increased burden on the protein homeostasis machinery.^22^ Indeed, genetic screens of transformed cells, including Ras‐transformed and triple‐negative breast cancer cells, revealed a strong dependency on the 26S/20S proteasome ratio.^23^, ^24^, ^25^ Furthermore, we have reported that the depletion of 19S RP subunits reduces the 26S proteasome level, lowers the cellular 26S/20S ratio, and severely compromises cancer cell viability.^4^ In contrast, normal human fibroblasts are resistant to the same type of 26S proteasome depletion. In advancing cancer therapy, it is important to investigate the tumor cell lines addicted to high 26S proteasome levels in animal models. Here, we describe a set of experiments to examine the growth of two aggressive cell lines, MDA‐MB‐231, a triple‐negative breast tumor cell line (TNBC), and OVCAR8, a high‐grade ovary adenocarcinoma, to generate xenograft mouse models. We found that PSMD1 depletion robustly inhibited the growth of these tumor cell lines. We also show that the PSMD1 depletion strategy effectively reduces primary ovarian cancer growth in mice.

## 2 MATERIALS AND METHODS

### 2.1 Cell lines

MDA‐MB‐231 cell line represents TNBC. OVCAR‐8 represents high‐grade serous ovarian cancer (HGSOC). Both cell lines were obtained from the National Cancer Institute collection (NCI‐60) and cultured in RPMI medium, supplemented with 10% fetal bovine serum (Invitrogen Life Technologies), 2 mM glutamine, and 1% penicillin–streptomycin (Biological Industries). Cells were maintained in a humidified atmosphere of 5% CO_2_ at 37°C.

We generated a lentiviral vector, pLenti6‐Luciferase (pLenti6‐Luc), expressing the firefly luciferase to assess tumor progression. pLenti6‐Luciferase expression lentiviral vector was used to transduce MDA‐MB‐231 cells. Stably transduced cells were selected using blasticidin. We termed these cells MDA‐MB‐231‐Luc. The luciferase assay verified the luciferase activity of MDA‐MB‐231‐Luc cells.

### 2.2 Lentiviral production

Lentiviral vectors were generated to express shRNAmir (Open Biosystems, Thermo Scientific) either constitutively (pGIPZ plasmid) or in a Tet‐inducible (TRIPZ plasmid) manner to downregulate PSMD1 levels. HEK‐293T cells transfected (calcium phosphate method) with the lentivirus expression vectors (20 μg per 15 cm plate), and the helper components pCMV‐VSV‐G (5 μg) and pCMV ΔR8.9 (15 μg) to produce virions.

Viral supernatants were collected after 48 and 72 h and filtered through a 0.45 μm filter to remove cell debris. Viral particles were concentrated by two consecutive spins of ultracentrifugation at 70,000 *g* for 2 h and resuspended in Hank's Balanced Salt Solution (HBSS). To determine the multiplicity of infection, 10‐fold serial dilutions were made of the lentiviral preparation in PBS.^26^ HEK293T cells (10^5^ per well of the 24‐well plate) were infected with 20 μL of each viral dilution, together with polybrene (8 μg/mL). Cells were grown for 48 h for maximal fluorescent signal (GFP is expressed from the pGIPZ vectors used in this study). Biological titer (BT) was calculated BT = TU/mL, (TU is transducing units) according to the following formula: TU/μL = (*P* × *N*/100 × *V*) × 1/DF, where *P* is the % GFP+ cells, *N* is the number of cells at the time of transduction, *V* is the volume of dilution added to each well, and DF is the dilution factor.

### 2.3 Induction and validation of PSMD1 knockdown in vitro

Cells were infected with Tet‐inducible TRIPZ lentivirus particles and selected with 2 μg/mL puromycin. For inducing shRNA expression to knockdown PSMD1 in vitro, cells were treated with 1 μg/mL doxycycline (dox) for 4–5 days. Protein extraction and immunoblot analysis were done as previously described.^4^ Cell proliferation was analyzed using the XTT assay (Biological Industries) and spectrophotometrically quantified. Cells were also photographed in situ by the Incucyte® SX1 live‐cell analysis system (Sartorius), at 10× magnification, with 25 images per well every 12 h. The Incucyte analysis software was used to calculate percent confluence. Cell cycle analysis was done by flow cytometry as previously described.^4^

Generation of tumor xenografts in mice: All animal studies were preapproved by the Weizmann Animal Care and Use Committee (IACUC) and the Institute's Review Board (IRB) for working with human‐originated models (IACUC Protocol number: 34840317‐2). Female nude mice HsdHli:CD1‐Foxn1nu (6–7 weeks old) were obtained from Envigo. All mice were maintained in specific pathogen‐free, temperature‐controlled (22°C ± 1°C) mouse facility on a 12/12 h light/dark cycle, Animals were fed a regular chow diet et libitum and allowed to acclimate before the start of experiments.

Cells were subcutaneously injected into the right back of each mouse. Routinely, 50 μL of cell suspension per mouse was injected under sterile conditions using 27G needle‐equipped syringes. Digital caliper measurements followed tumor size. Tumor volume was calculated as X2Y/2 (X is the smallest tumor dimension).

To induce shPSMD1 expression in vivo, we used dox (Glentham Life Sciences) (1 mg/mL) in drinking water protected from light, starting from day 5 after injection. Due to the bitter taste of dox, 1% sucrose was added to the drinking water. The drinking water was changed by a fresh one every 3 days.

For intratumor lentiviral injections, HsdHli:CD1‐Foxn1nu female nude mice (7 weeks old) were injected with MDA‐MB‐231 or OVCAR8 cells. Lentivirion constitutively expressing shPSMD1 and GFP was injected when the tumors reached ~20 mm^3^ sizes (about 10 days after injection of cells). For controls, we injected either lentivirions constitutively expressing non‐silencing shRNA sequence and GFP, or the physiological HBSS solution. 2–10 × 10^6^TU were usually used for a single injection.

### 2.4 Bioluminescent in vivo imaging

The growth of the subcutaneously implanted cells was examined twice a week by bioluminescent image analysis using the IVIS SPECTRUM (Caliper Life Sciences) imaging system. As light is directly emitted by tumor cells, bioluminescence is a highly specific and sensitive methodology for tumor detection and follow‐up over time. d‐luciferin (500 μg in 100 μL of PBS) (Regis Technologies) was injected into the abdominal cavities of the mice. Mice were anesthetized with a mixture of oxygen and isoflurane following imaging. Monitoring started 10 min after injection of d‐luciferin. The exposure time was set to 1 s. The parameters of exposure and imaging were kept constant for each measurement during the study. Image signals were analyzed using the Living Image® software (Xenogen). The growth of the subcutaneously implanted cells was depicted by a tumor‐growth curve (average radiance [P/s/cm^2^/sr]) vs. time in days post‐implantation).

### 2.5 Fluorescent imaging

Since RFP is a marker of LV‐shPSMD1 cassette‐inducible expression, we performed fluorescence imaging in mice injected with LV‐shPSMD1 cassette‐bearing cells, either treated or not treated with dox. Since GFP is a marker of successful transduction by the constitutive LV‐shPSMD1, we performed fluorescence imaging in mice injected with LV‐shPSMD1 virions.

### 2.6 Generation of ovarian patient‐derived ovarian cancer xenograft (PDX) xenografts and intratumor LV‐shPSMD1 injections

For ovarian PDX experiments, we used a metastatic high‐grade ovarian carcinoma model from the Jackson Laboratory (Model ID TM00327; http://tumor.informatics.jax.org/mtbwi/pdxDetails.do?modelID=TM00327), and implanted in NSG mice (Jackson Laboratories). NSG mice are the most highly immunodeficient mice and the model of choice for cancer xenograft modeling. Briefly, following euthanasia, tumors were removed from donor mice and cut into small fragments of about 2 mm in diameter. Recipient NSG mice were anesthetized with isoflurane. A small pouch was made in the lower back of the mouse, and a tumor fragment was inserted into the pouch. The wound was closed using a surgical clip. Clips were removed 4–5 days after surgery. Tumor volume was monitored by caliper measurements and calculated as above. Since PDX models are characterized by high heterogeneity, tumors appeared after a variable period following implantation (7–9 weeks) and therefore were treated in cohorts. Either constitutively active LV‐shPSMD1 or irrelevant sequence‐based virions (~5 × 10^6^TU) were intratumorally injected each time. For each group of treatment, 9–11 mice were used. The volume of the tumors was measured by caliper measurements as described above.

## 3 RESULTS

### 3.1 MDA‐MB‐231 and OVCAR8 cells are highly susceptible to PSMD1 depletion

**FIGURE 1:**
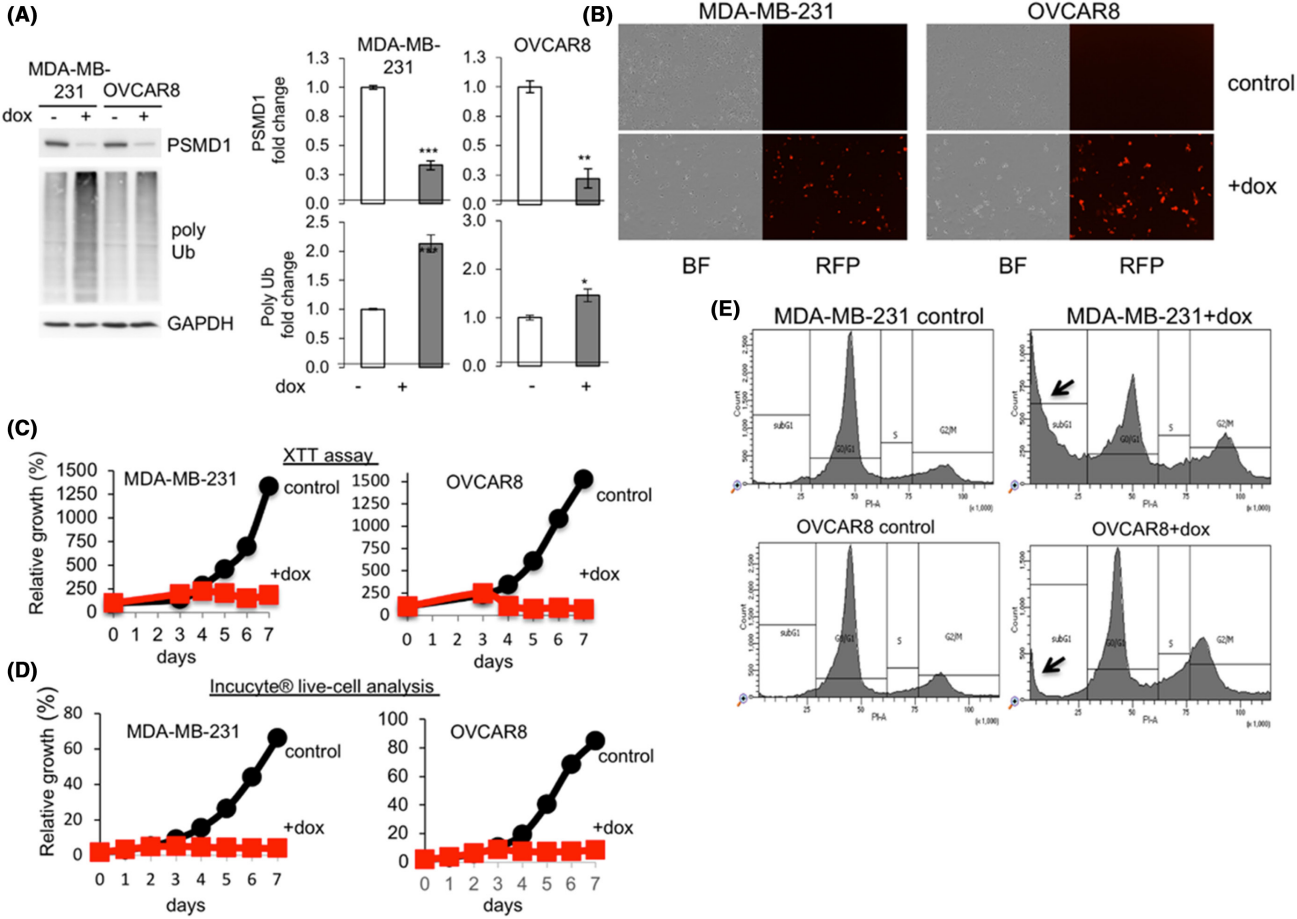
Triple‐negative breast cancer MDA‐MB‐231 and the high‐grade ovary adenocarcinoma OVCAR8 cell lines are addicted to high 26S proteasome levels. (A) The levels of PSMD1 and polyubiquitinated proteins in MDA‐MB‐231 and OVCAR8 cells harboring a doxycycline‐inducible PSMD1 shRNA before and after doxycycline (dox) treatment. The extracts were immunoblotted with PSMD1, ubiquitin, and GAPDH antibodies. The quantifications of three replicates are shown. (B) Microscopic images of control and dox‐treated microscopic images of cells described under panel A, shown by bright field (BF) and RFP expression. RFP is expressed only when doxycycline induction is effective and thus monitors shRNA expression. (C) Growth of MDA‐MB‐231 and OVCAR8 cells expressing dox‐inducible PSMD1 shRNA was analyzed using the XTT assay (*N* = 3). (D) The results obtained by the XTT, cell viability assay, were confirmed by in situ cell monitoring using the Incucyte® SX1 live‐cell analysis system (*N* = 3). In (C) and (D) standard error means are lower than 5%, and *p* < 0.001 from Day 4 and on. (E) Cell death was quantified by measuring the subG1 fraction (marked by an arrow) by FACS analysis.

Previously, we have reported that PSMD1 depletion reduces the 26S/20S ratio accompanied by massive death of a number of aggressive tumor cell lines.^4^ We compared here the response of MDA‐MB‐231, a TNBC, and OVCAR8, high‐grade ovary adenocarcinoma, to PSMD1 depletion. To this end, we transduced the cells with a lentivector expressing PSMD1 shRNA and RFP reporter gene under a dox‐inducible promoter. Dox treatment resulted in a marked reduction in the level of PSMD1, a 19S RP subunit, in both cell lines (Figure 1A). Depleting PSMD1 resulted in the accumulation of polyubiquitinated proteins, suggesting that the 26S proteasome activity was reduced. Microscopic visualization revealed a high level of RFP expression upon dox treatment accompanied by much lower cell number (Figure 1B). Cell viability was monitored by XTT (Figure 1C) and live cell analysis (Figure 1D) for7 days. The growth of dox‐treated cells was severely compromised upon shPSMD1 expression. FACS analysis revealed massive MDA‐MB‐231 cell death, as evident by the high level of subG1 fraction and G2M accumulation (Figure 1E). Under these conditions, OVCAR8 cells were mainly G2/M blocked. These data suggest that MDA‐MB‐231 and OVCAR8 cells are highly susceptible to PSMD1 depletion.

### 3.2 Treating MDA‐MB‐231 xenograft mice with inducible shPSMD1

Xenografts of human tumors in mice are important preclinical in vivo test models for the evaluation of response to new therapy. In optimizing the system, we inoculated increasing numbers of control and the shPSMD1 positive but not induced MDA‐MB‐231 cells. We compared the growth kinetics of mice inoculated with control MDA‐MB‐231 cells with that inoculated with an inducible shPSMD1 cassette. The results show that subcutaneous tumors were formed at the injection site in all the mice inoculated with either 4 or 8 × 10^6^ cells. Some mice inoculated with 2 × 10^6^ cells did not develop tumors. However, tumors inoculated with 8 × 10^6^ cells developed too rapidly. Therefore, the amount of 4 × 10^6^ cells was chosen as optimal for further experiments (Figure S1). In addition, these results revealed that both the control and the genetically manipulated cells grew with similar kinetics. Thus, there was no significant leakiness from the inducible LV‐shPSMD1 cassette to affect tumor growth.

**FIGURE 2:**
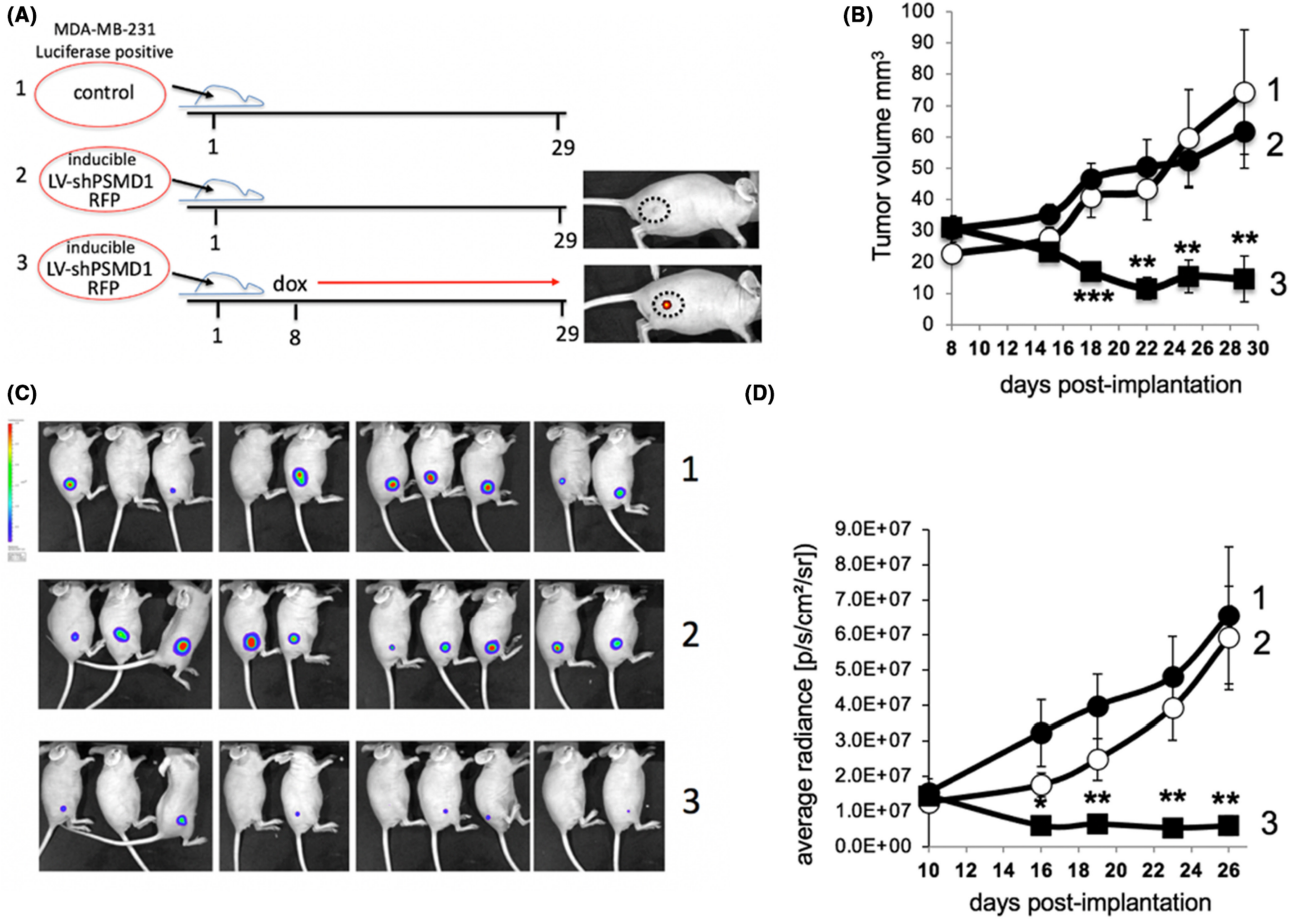
shPSMD1 depletion effectively reduced the tumor size in a xenograft model. (A) Schematic presentation of the experimental design of the three treated mice groups labeled 1–3 in all the panels. Ten female nude mice (HsdHli:CD1‐Foxn1nu; 6–7 weeks old) were obtained from Envigo per group. The red line indicates the dox‐dependent induction of expression, as visualized by live imaging (cf. the mice of Group 2 to Group 3). Either control or inducible shPSMD1 cassette harboring cells (4 × 10^6^) were subcutaneously injected into the right back of each mouse on Day 1. To induce shPSMD1 expression, mice were treated with doxycycline (1 mg/mL) in drinking water starting from Day 5 after injection. (B) Caliper measurements examined tumor growth rate. Tumor volume was calculated as X2Y/2 (X is the smallest tumor dimension). (C) Statistical calculation of the average tumor volume, on Day 29, by boxplot demonstration. *p < 0.05, **p < 0.01. (D) Luciferase live imaging of the three groups of mice was conducted at the end of the experiment (Day 26 after injection of tumor cells) using IVIS SPECTRUM. *p < 0.05, **p < 0.01, ***p < 0.001.

Next, for reliably monitoring tumor development and sizes both cells were transduced to express the Luciferase reporter gene. We inoculated 4 × 10^6^ MDA‐MB‐231 cells in the xenograft model. Three groups were used; in Group 1, control cells were injected, and in Groups 2 and 3, cells were first transduced with a cassette expressing in an inducible manner RFP and shPSMD1. Dox was added to the drinking water of Group 3 (Figure 2A). The expression of RFP monitored the efficient effect of dox in the induction of transcription. Tumor size measurement of the mice revealed a robust reduction in the tumor volume of the group treated with dox (Figure 2B,C). Bioluminescent imaging revealed a similar decrease in tumor size as shown per mouse (Figure 2D). These data suggest that the MDA‐MB‐231 cells are highly susceptible to PSMD1 depletion in a xenograft mice model.

### 3.3 Treating MDA‐MB‐231 xenograft mice with the intratumor injection of LV‐shPSMD1

**FIGURE 3:**
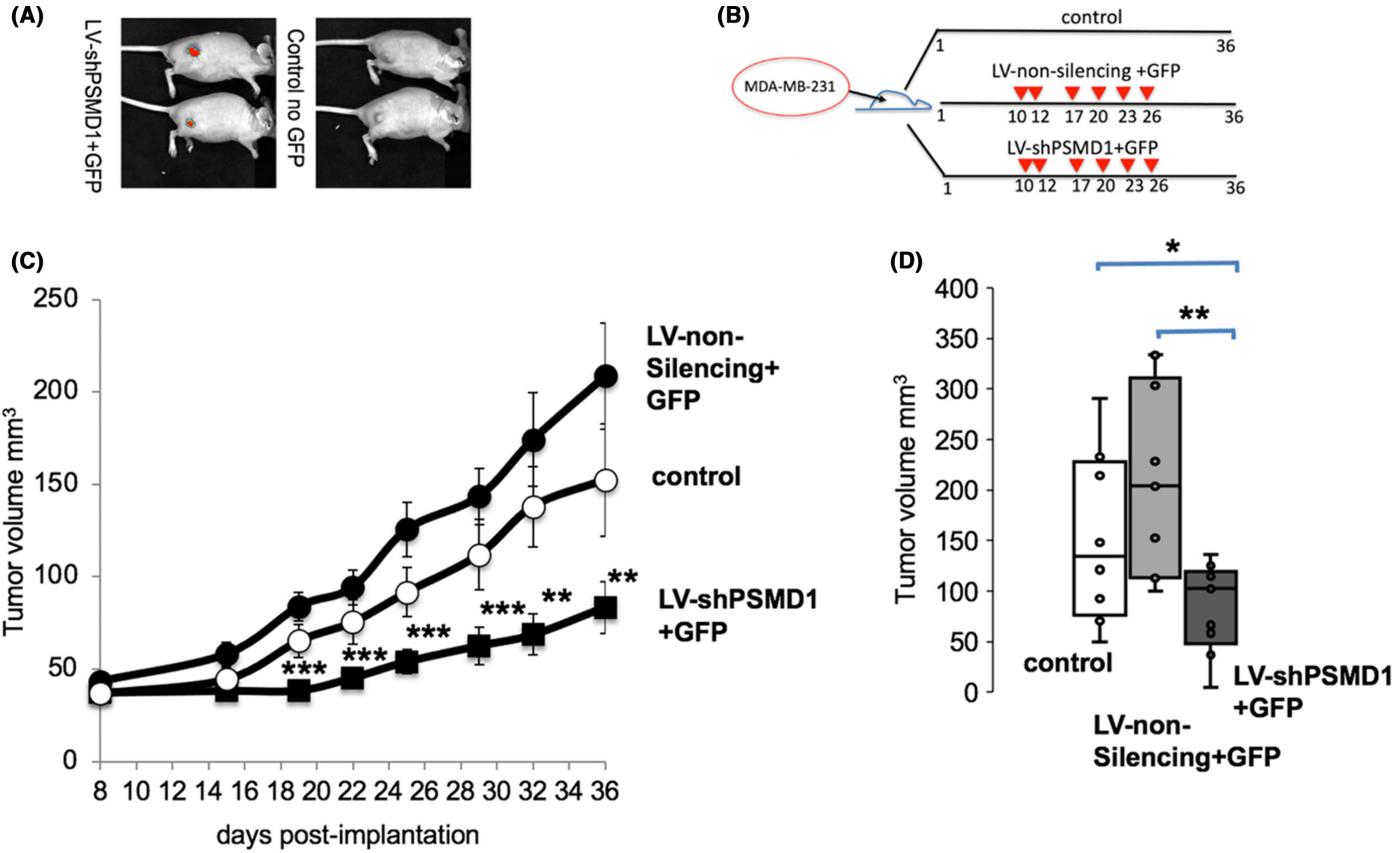
Intratumor LV‐shPSMD1 injection reduced tumor size. (A) GFP expression (the red spot) in the tumors after intratumor injection of the lentivector expressing GFP. (B) Schematic presentation of the design of the experiments. MDA‐MB‐231 cells (4 × 10^6^) were injected subcutaneously into the right back of each mouse on Day 1. Group 1 was intratumorally injected with the control lentivector, and Group 2 with LV‐shPSMD1 and GFP (2–10 × 10^6^TU). The second control group was injected with HBSS physiological solution only. The injection was repeated on the days labeled by an arrow. The expression of GFP, as detected by live imaging, validated the presence of the LV‐shPSMD1 in the tumor site. (C) Caliper measurements examined tumor volume. Tumor volume was calculated as X2Y/2 (X is the smallest tumor dimension). (D) The statistically calculated average tumor size by boxplot on Day 36. *p < 0.05, **p < 0.01, ***p < 0.001.

MDA‐MB‐231 xenografts were established by subcutaneous injections of 4 × 10^6^ MDA‐MB‐231 cells into the right back of each 7‐week‐old female nude mice (HsdHli: CD1‐Foxn1nu). The control group was not lentivector transduced, but intratumor injected with HBSS physiological solution. The mice of the second control group were repeatedly intratumor injected with lentivector constitutively expressing GFP and non‐silencing shRNA sequence. To validate lentivector transduction by intratumor injection, we examined GFP expression by live imaging (Figure 3A). The results demonstrate efficient intratumor lentivector transduction. Subcutaneous injections of 4 × 10^6^ MDA‐MB‐231 cells into the right back of each 7‐week‐old female nude mice (HsdHli: CD1‐Foxn1nu). The control group was not lentivector transduced. The mice of the second control group were repeatedly intratumor injected with lentivector constitutively expressing lentivector lacking the shPSMD1 silencing cassette. The experimental group was injected with a lentivector constitutively expressing shPSMD1 and GFP at the indicated time points (Figure 3B). The results show that the injection of LV‐shPSMD1 significantly reduced tumor growth in mice (Figure 3C,D). These data suggest that intratumor injection of lentivector expressing shPSMD1 compromised tumor development in MDA‐MB‐231 xenograft mice.

### 3.4 Treating OVCAR8 xenograft mice with inducible shPSMD1

To optimize the number of OVCAR8 cells per mouse increasing number of cells were subcutaneously injected, and the tumor size was evaluated throughout the experiment (Figure S2). The growth rate of control OVCAR8‐injected cells was compared with that of the OVCAR8 cells transduced with a lentivector expressing the inducible shPSMD1 cassette but were not dox treated. All cells grew with similar kinetics. Based on this experiment, we injected 8 × 10^6^ cells in the follow‐up experiments.

**FIGURE 4:**
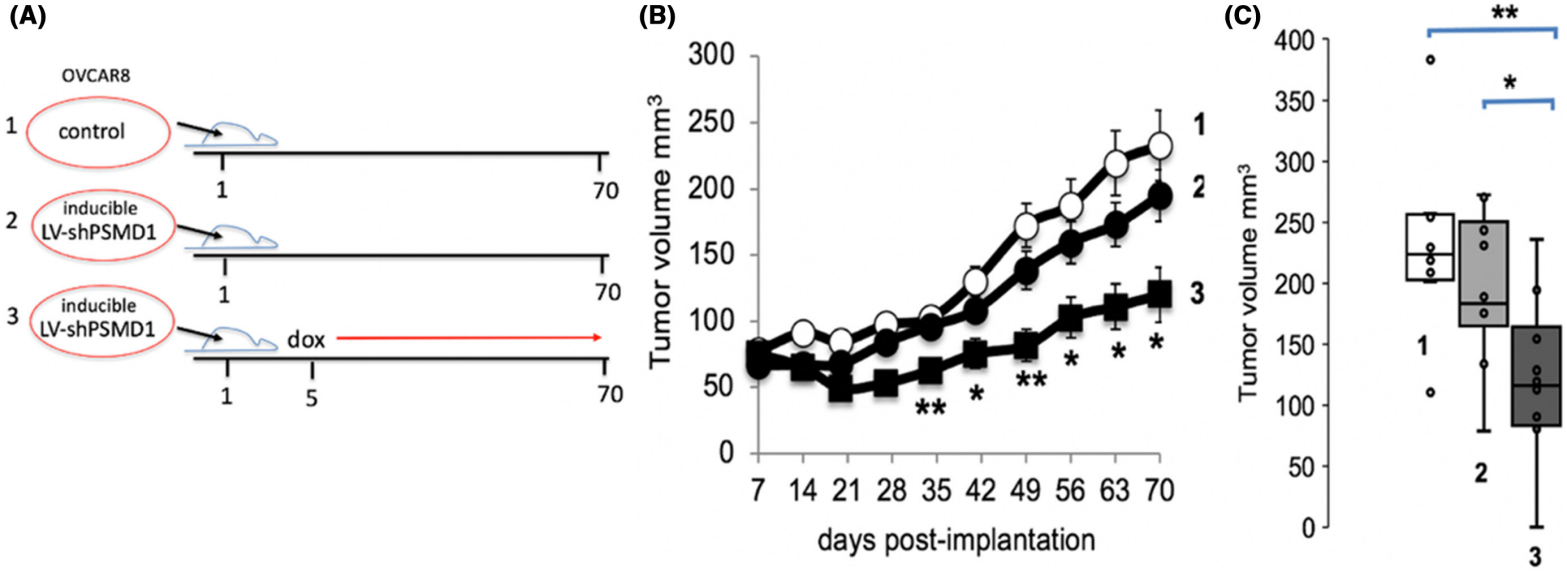
LV‐shPSMD1 expression effectively reduced the OVCAR8 tumor size in the xenograft mice model. (A) Schematic presentation of the experimental design of the three different treated mice groups, numbered 1–3 in all the panels. The red line indicates the dox‐dependent induction. (B) Either control or inducible LV‐shPSMD1 cassette cells (8 × 10^6^) were subcutaneously injected into the right back of each mouse on Day 1. To activate LV‐shPSMD1, we used doxycycline (dox) supplementation (1 mg/mL) in drinking water starting from Day 5 after injection. Caliper measurements examined tumor growth. (C) The statistically calculated average tumor size by boxplot on Day 70. *p < 0.05, **p < 0.01.

Next, we generated xenograft mice implanted with either control OVCAR8, the control mice, or OVCAR8 transduced with a lentivector carrying the inducible shPSMD1 cassette. Half of the latter group was treated with dox (1 mg/mL) in drinking water, starting from Day 5 after injection (Figure 4A). The drinking water was changed every 3 days. In the two xenograft control groups, the group that was injected with the control OVCAR8 cells and the group injected with OVCAR8 cells that were transduced with lentivector carrying the inducible shPSMD1, but remained uninduced, showed a similar rate of tumor growth with the latter group showing a slightly lower rate (Figure 4B), possibly because of a certain level of shPSMD1 expression. The tumor development of the dox‐induced group treated to express shPSMD1 was significantly lower from Day 35 to the end of the experiment, Day 70. On Day 70 post‐injection of the tumor cells, the tumor volume of the dox‐induced group was significantly lower than that of the two control groups (Figure 4C). These data suggest that the depletion of PSMD1, a 19S proteasome regulatory complex component, slows OVCAR8 tumor growth in the xenograft mouse model.

### 3.5 Treating the mice bearing ovarian cancer PDX xenograft with LV‐shPSMD1

**FIGURE 5:**
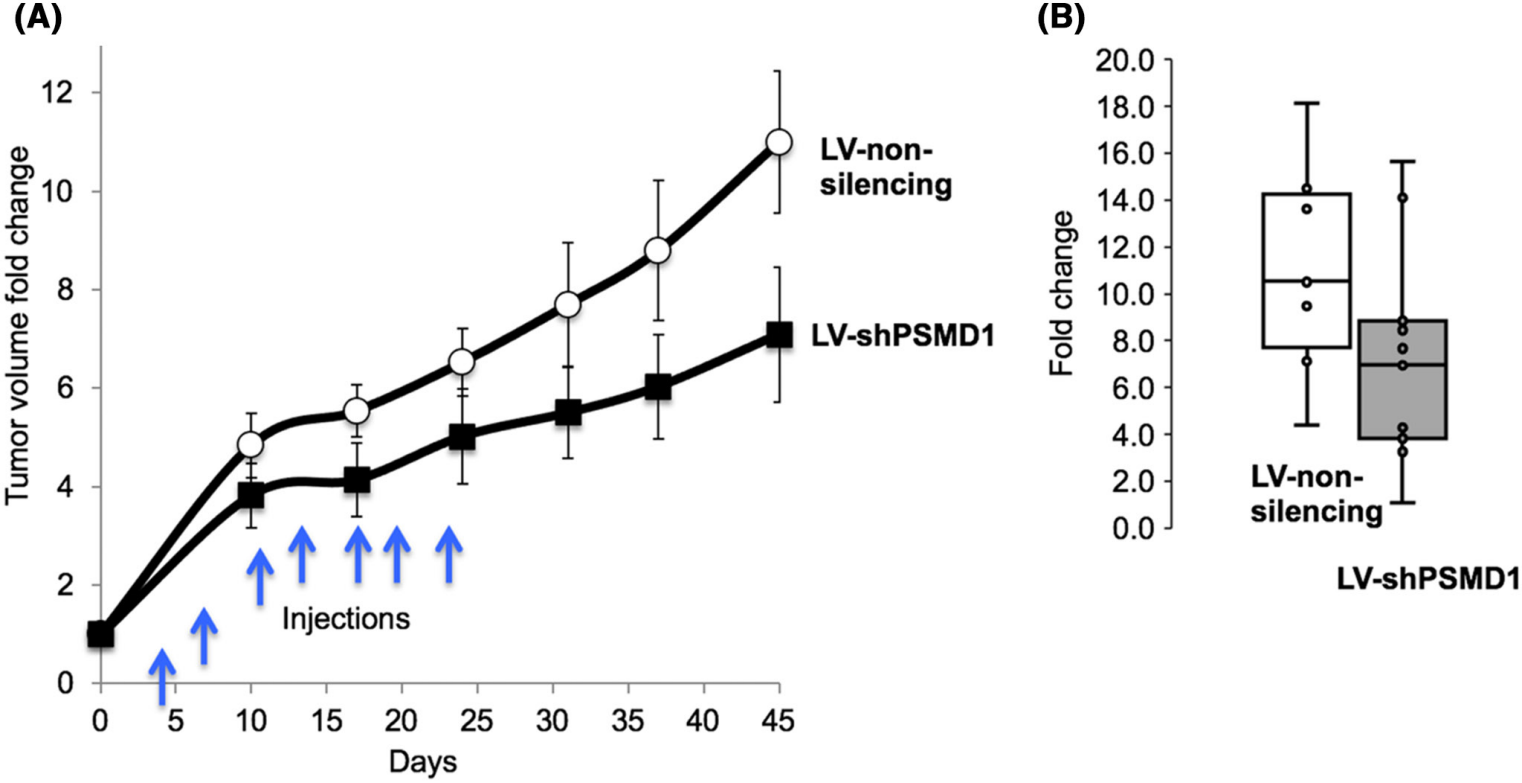
Treating the mice bearing PDX xenograft tumors with LV‐shPSMD1 injections. (A) NSG NSG mice were implanted with metastatic high‐grade ovarian carcinoma PDX model. Treatments started when the tumors reached ~20 mm^3^ sizes. Either constitutively active LV‐shPSMD1 or irrelevant sequence‐based virions (~5 × 10^6^TU) were injected each time (the blue arrows) intratumorally. The volume of the tumors was measured as described above. For each group of treatment, 9–11 mice were used. (B) The boxplot's statistically calculated average tumor size is indicated in the right panel. The growth rate with the time of the tumor is lower in the treatment compared to the control (*p* = 0.0001). The *p*‐value of the growth rate is of the interaction term in a mixed effect linear model with the time and the interaction between the time and the treatment as the fixed effects, and the mouse as the random effect.

Next, we used the patient‐derived xenograft (PDX) model to treat ovarian cancer by the PSMD1 depletion strategy. The lentivector transduction for shPSMD1 expression and control shRNA expression was started when tumors achieved a volume of ~20 mm^3^. Since PDX models are characterized by high heterogeneity, tumors reached this volume after a variable time following implantation (7–9 weeks) and therefore were treated in cohorts. Lentivirus inoculums of ~5 × 10^6^TU were intratumorally injected on the indicated days (twice a week, a total of seven times) (Figure 5A). Tumor fold change is plotted versus the tumor fold change over time from the initial injection (Day 0). The growth rate of the primary ovarian tumor was slower in the shPSMD1‐treated tumor reaching maximal difference on the last day of the experiment, Day 45 (Figure 5B). Comparing coefficients in regression analysis showed that intratumor injection of LV‐shPSMD1 significantly reduces the development of PDX tumors in mice, supporting the possibility of implementing the strategy of depletion of the components of the 19S proteasome regulatory complex in the therapy of aggressive cancers.

## 4 DISCUSSION

Here we show that the depletion of PSMD1, a component of the proteasomal 19S regulatory complex, is a practical approach to reducing tumor size in the mice xenograft model. The growth of two rather aggressive tumor cell lines, MDA‐MB‐231, a TNBC, and OVCAR8, a high‐grade ovary adenocarcinoma, was significantly reduced upon expression of shPSMD1. We further demonstrated that PDX of primary ovarian cancer could be treated with a lentivector expressing shPSMD1.

The non‐ATPase components of the proteasomal 19S regulatory complex (RP) of the PSMD group are highly expressed in different tumors and are proposed to be a good target for cancer therapy.^4^, ^27^ In pancreatic ductal adenocarcinoma patients, high levels of PSMD6, PSMD9, PSMD11, and PSMD14 are associated with a lower rate of survival.^28^ The levels of PSMD1, PSMD2, PSMD3, PSMD7, PSMD10, PSMD12, and PSMD14 are high in breast cancer tissue compared to normal tissues.^29^ Here again, the increased levels correlate with poor prognoses in breast cancer patients.

PSMD1 was proposed to facilitate the progression of lung adenocarcinoma.^30^ In urothelial bladder carcinoma, PSMD2, PSMD3, PSMD4, PSMD8, and PSMD11 genes are overexpressed.^31^ High levels of PSMD1 and PSMD3 mRNA were observed in CML patients, especially those in the blast phase.^27^ PSMD1 and PSMD3 depletion induced apoptosis in CML cells. All these findings suggest that PSMD proteins are a good target in the therapy of various cancer types, as has been reported at the level of cell lines.^4^

PSMD1 depletion gives rise to the lower level of 26S PC, the major and critical player in cell growth and survival. The observation that tumors overexpress PSMD proteins suggests that tumor cells are contained with high 26S PC levels. This has been demonstrated by Ras transformation of MCF10A cells, where the accumulation of the proteasomal subunits with concomitant increase at the 26S PC level was reported.^4^ Furthermore, reducing the 26S/20S ratio severely compromises tumor cell growth with only a minor effect on normal cells.^4^, ^27^ We have reported that over 20 different tumor cell lines of varying origins did not survive reduction at the level of the 26S proteasome. The more aggressive the tumor cells are, the more susceptible they are to a low 26S/20S ratio. These include the aggressive ovarian and TNBC cell lines.^4^ The findings reported here not only lend further support but also provide in vivo evidence for this notion. However, in the xenograft models, the growth of the MDA‐MB‐231 cells is dramatically suppressed, while the OVCAR8 was less responsive. One reason relies on the slower growth kinetic of the OVCAR8 tumors. It took 70 days to reach the tumor size of MDA‐MB‐231 xenografts on Day 36. Additionally, as demonstrated in Figure 1A, the accumulated polyubiquitinated proteins in response to PSMD1 knockdown are lower in OVCAR8 cells and therefore experience milder proteotoxic stress.

Depletion of PSMD1 reduces the 26S/20S ratio. Several small molecules were reported to reduce the 26S/20S ratio.^32^, ^33^ One of these small molecules was effective in reducing multiple myeloma cell growth in the culture and xenograft model.^34^ Small molecules are thus an attractive option to treat cancer by targeting the 26S complex. However, we must remember that the shRNA or siRNA strategy has a lower probability of off‐target activity. Additionally, since shRNA targeted against several 19S components effectively kills tumor cells,^4^ a combinatorial shRNA/siRNA treatment would dramatically reduce the development of tumor escape mutants.

## AUTHOR CONTRIBUTIONS

**Julia Adler:** Data curation (supporting); formal analysis (supporting); investigation (lead); methodology (lead). **Roni Oren:** Formal analysis (supporting); investigation (supporting); methodology (equal). **Yosef Shaul:** Conceptualization (equal); data curation (equal); formal analysis (equal); funding acquisition (equal).

## CONFLICT OF INTEREST STATEMENT

The authors declare no conflict of interest.

## Supporting information


**Data S1:** Supporting Information

## Data Availability

Data sharing is not applicable to this article as no new data were created or analyzed in this study.
