# Supplementary material for: Depleting the 19S proteasome regulatory PSMD1 subunit as a cancer therapy strategy
Source: Cancer Med. 2023 Mar 19;12(9):10781–90. doi: 10.1002/cam4.5775 (PMC10225209; doi:10.1002/cam4.5775)
Supplement: Supplementary file 1 — Data S1: Supporting Information [file CAM4-12-10781-s001.docx]

**Supplementary Materials**

**Depleting the 19S proteasome regulatory PSMD1 subunit as a cancer therapy strategy**

Julia Adler^1^, Roni Oren^2^ and Yosef Shaul^1*^

^1^ Department of Molecular Genetics, Weizmann Institute of Science, Rehovot 76100, Israel

^2^ Department of Veterinary Resources, Weizmann Institute of Science, Rehovot 76100, Israel

***** Correspondence: Address: Department of Molecular Genetics, Weizmann Institute of Science, Herzl St 234, Rehovot 76100, Israel, yosef.shaul@weizmann.ac.il; Tel.: +972 8 934-2320


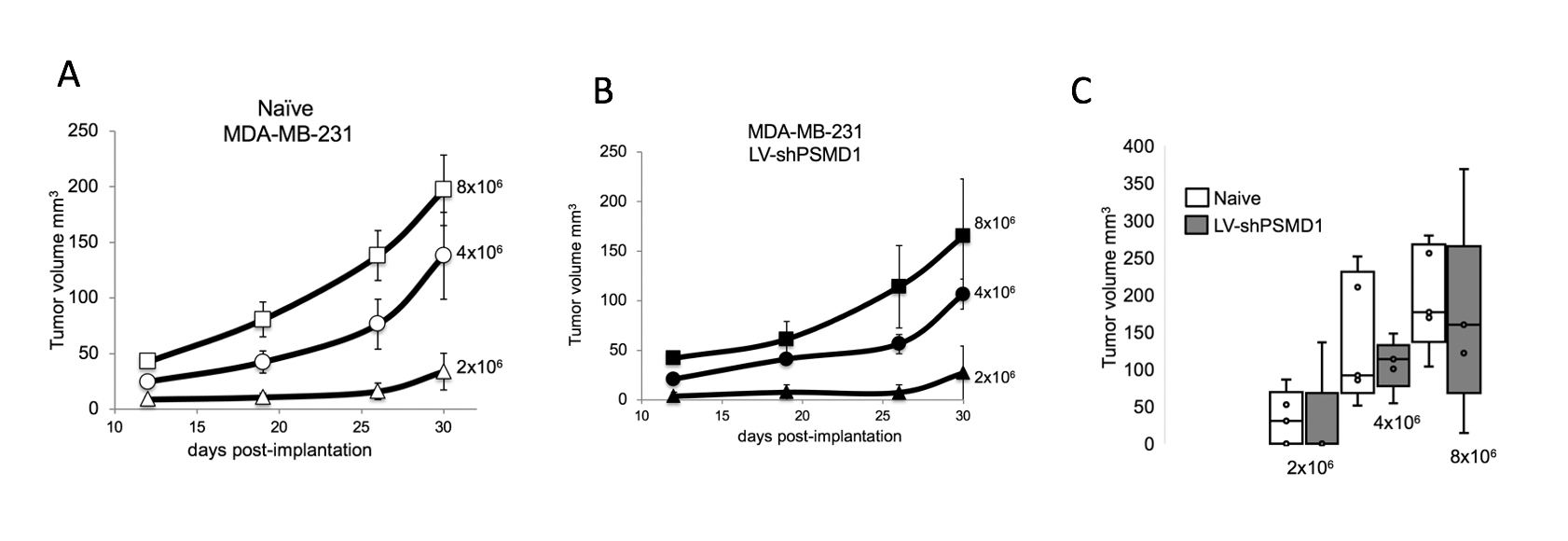


**Fig S1: Xenograft experiment to** **assess the number of MDA-MB-231cells to induce tumors.**

The naïve MDA-MB-231 cells (A) and cells harboring an inducible LV-shPSMD1 cassette (B) were injected subcutaneously with the indicated number of cells at day 1. Mice were maintained without doxycycline supplementation (no induction of shPSMD1 expression). Tumor growth was examined by caliper measurements. Tumor volume was calculated as X^2^Y/2 (X is the smallest tumor dimension). C) The statistical calculated average tumor size by boxplot at day 30. Both naïve and the shPSMD1 cassette harboring cells formed tumors and grew with similar kinetics in a dose-dependent manner. Based on this experiment we injected 4 x 10^6^ cells in the follow up experiments.

-
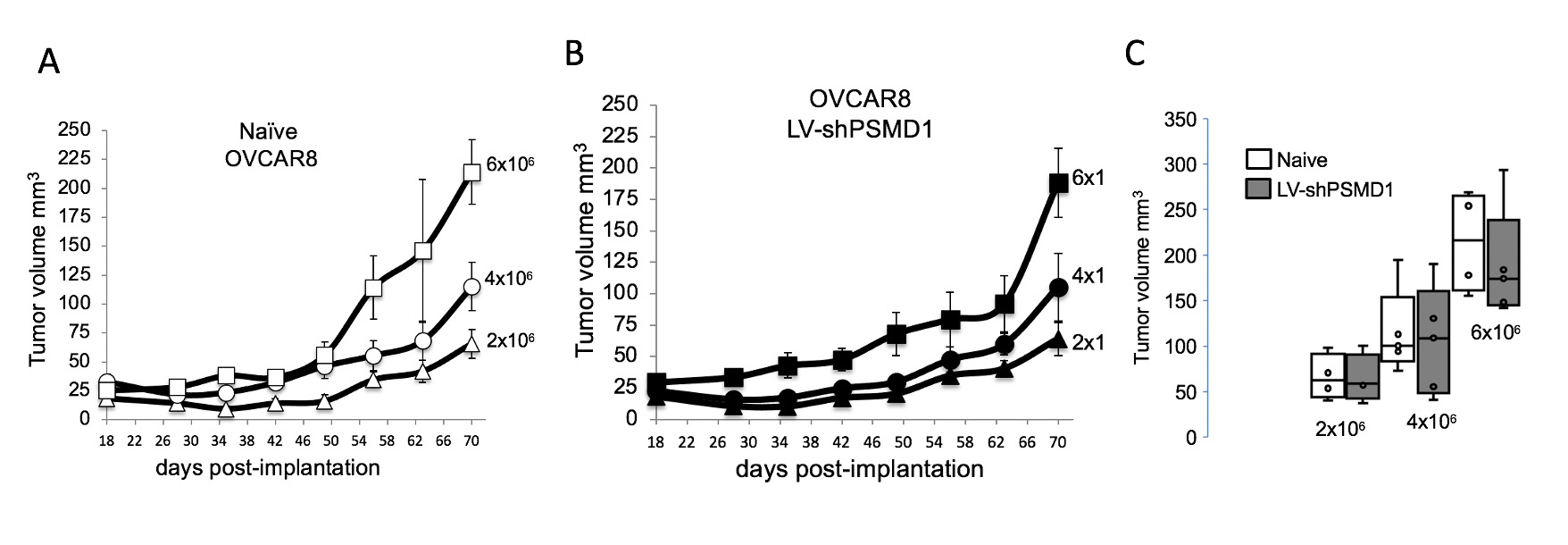


Figure S2: **optimizing the number of** **OVCAR8 xenograft cells to induce tumors.**

- The naïve OVCAR8 cells (A) and cells harboring an inducible LV-shPSMD1 cassette (B) were injected subcutaneously with the indicated number of cells on day 1. Six groups of five mice were used for the study. Mice were maintained without doxycycline supplementation. Tumor size was measured by caliper. Both naïve and inducible LV-shPSMD1 cassette harboring cells formed tumors. C) The statistical calculated average tumor size by boxplot on day 70. The tumor grew with similar kinetics in a dose-dependent manner. Thus, there is no significant leakiness from the inducible LV-shPSMD1 cassette without induction with doxycycline.
